# The effects of occipital and parietal tDCS on chronic visual field defects after brain injury

**DOI:** 10.3389/fneur.2024.1340365

**Published:** 2024-02-14

**Authors:** Lorenzo Diana, Carlotta Casati, Lisa Melzi, Stefania Bianchi Marzoli, Nadia Bolognini

**Affiliations:** ^1^Laboratory of Neuropsychology, Department of Neurorehabilitation Sciences, IRCCS Istituto Auxologico Italiano, Milan, Italy; ^2^Neuro-Ophthalmology Center and Ocular Electrophysiology Laboratory, IRCCS Istituto Auxologico Italiano, Milan, Italy; ^3^Department of Psychology, University of Milano-Bicocca and NeuroMI, Milan, Italy

**Keywords:** visual field defects, hemianopia, compensatory training, multisensory training, transcranial direct current stimulation, tDCS

## Abstract

**Introduction:**

Homonymous visual field defects (HVFDs) following acquired brain lesions affect independent living by hampering several activities of everyday life. Available treatments are intensive and week- or month-long. Transcranial Direct current stimulation (tDCS), a plasticity-modulating non-invasive brain stimulation technique, could be combined with behavioral trainings to boost their efficacy or reduce treatment duration. Some promising attempts have been made pairing occipital tDCS with visual restitution training, however less is knows about which area/network should be best stimulated in association with compensatory approaches, aimed at improving exploratory abilities, such as multisensory trainings.

**Methods:**

In a proof-of-principle, sham-controlled, single-blind study, 15 participants with chronic HVFDs underwent four one-shot sessions of active or sham anodal tDCS applied over the ipsilesional occipital cortex, the ipsilesional or contralesional posterior parietal cortex. tDCS was delivered during a compensatory multisensory (audiovisual) training. Before and immediately after each tDCS session, participants carried out a visual detection task, and two visual search tasks (EF and Triangles search tests). Accuracy (ACC) and response times (RTs) were analyzed with generalized mixed models. We investigated differences in baseline performance, clinical-demographic and lesion factors between tDCS responders and non-responders, based on post-tDCS behavioral improvements. Lastly, we conducted exploratory analyses to compare left and right brain-damaged participants.

**Results:**

RTs improved after active ipsilesional occipital and parietal tDCS in the visual search tasks, while no changes in ACC were detected. Responders to ipsilesional occipital tDCS (Triangle task) had shorter disease duration and smaller lesions of the parietal cortex and the superior longitudinal fasciculus. On the other end, on the EF test, those participants with larger damage of the temporo-parietal cortex or the fronto-occipital white matter tracts showed a larger benefit from contralesional parietal tDCS. Overall, the visual search RTs improvements were larger in participants with right-sided hemispheric lesions.

**Conclusion:**

The present result shows the facilitatory effects of occipital and parietal tDCS combined with compensatory multisensory training on visual field exploration in HVFDs, suggesting a potential for the development of new neuromodulation treatments to improve visual scanning behavior in brain-injured patients.

## 1 Introduction

Homonymous visual field defects (HVFDs) following acquired brain lesions impact the survivors’ functional recovery and independent living by hampering several activities of everyday living, such as reading, finding objects in the surrounding space, navigating the space (i.e., bumping into people or getting lost), and driving a car (1). These impairments can lead to anxiety and depressive symptoms, affecting patients’ quality of life (2) and that of their caregivers.

Rehabilitative treatments for VFDs primarily involve restitutive and compensatory approaches (3, 4). Restitutive trainings include a variety of techniques aimed at reactivating lesioned, hypoactive visual networks, in order to restore visual functions, such as the enlargement of the defective visual field. Visual restoration therapy [VRT; (5, 6)], one of the most studied restitutive approaches, is a computerized training whereby the border zone of the scotoma is repeatedly stimulated with luminous stimuli, with the purpose of shifting the border area and reducing the blind hemifield. Despite some methodological controversies, a study with a large sample demonstrated the efficacy of VRT in terms of an enlarged visual field, enhanced detection abilities, and subjective improvements in everyday life (6). Instead, compensatory approaches train the patient to compensate for visual loss by developing more efficient eye movements and improving the speed and the accuracy of saccades toward the defective side of the visual field (7). This is achieved with paper-and-pencil or computerized visual scanning training that may include visual search and reading tasks [e.g., (8–10)]. Compensatory trainings can enhance search times and reduce the impact of HVFDs in the activities of daily living (11), and the improvements can persist for several years (12), even in absence of visual field enlargement (10).

Among compensatory trainings, multisensory, audio-visual trainings (AVTs) have been successfully employed to improve visual field scanning in acquired HVFDs (13–16). A recent review of 16 studies (17) confirmed that AVTs can improve visual information processing, as indexed by reduced search times in visual exploration tasks and reduced disability in activities of daily living [e.g., (13)], as well as an increased number of larger saccades in the blind hemifield (18). The effects of AVT are thought to be mediated by the activity of retino-collicular-extrastriate pathways, with a pivotal role of the multisensory neurons of the superior colliculus (SC) that integrate and amplify cross-modal signals (19). Combined with saccadic tasks, a repetitive, spatially and temporally congruent presentation of audio-visual stimuli can induce plastic changes in visual functions mediated by collicular activity, as recently demonstrated by Stein and Roland (20) in an animal model.

Irrespective of the type of approach, effective visual trainings are usually hospital-based, long (i.e., two weeks or longer), and intensive (i.e., multiple hours a day). Even though some logistical problems can be overcome [e.g., home-based multisensory training in (16) and https://osf.io/jmq86/ for an ongoing clinical trial], the idea of boosting brain plasticity by means of non-invasive brain stimulation (NIBS) to make rehabilitation faster and/or more effective has attracted some attention. Accordingly, visual trainings have been combined with non-invasive transcranial electrical stimulation techniques (tES) such as transcranial Direct Current Stimulation [tDCS; (21, 22)] or repetitive transorbital alternating current stimulation [rtACS; (23, 24)].

tDCS, in particular, relies on the application of a continuous current on the scalp and has been shown to modulate the excitability of the stimulated area, mostly on the motor cortex (25, 26), as well as on visual areas (27, 28), through long-term potential/depression mechanisms [e.g., (29)], inducing long-range effects on functionally connected networks [e.g., (30)]. Not surprisingly, tDCS has also been investigated as a tool to promote brain plasticity and recovery in diverse neuropsychiatric conditions (31), including acquired HVFDs, although to a much lesser extent (32, 33).

With respect HVFDs, most of the studies have applied anodal tDCS over the occipital cortex (to enhance brain plasticity in an excitatory fashion) in synergy with restitutive approaches designed to reactivate hypofunctioning populations of the visual network [(21, 34, 35); Matteo et al. (36); however, see (24, 37) for cathodal tDCS on contralesional occipital cortex]. For example, Plow et al. (22) showed, in chronic patients, an expansion of visual field, increased detection accuracy, and improved vision-related ADLs following a 3-month VRT (“border training”) combined with occipital anodal tDCS (compared to sham tDCS). More recently, Alber et al. (21) applied occipital tDCS to seven sub-acute patients undergoing VRT and observed an improvement in visual field sensitivity, which was higher than that of a control group who received a compensatory exploration training without tDCS. However, despite these promising results, the recent review by Perin et al. (32) concluded with a Level C recommendation for clinical application of tDCS for HVFDs.

To our knowledge, no studied attempted to combine tDCS with compensatory trainings in HVFDs patients, including those based on multisensory integration. As previously mentioned, these kind of approaches rely on the training of oculomotor functions in a context of audio-visual stimulation, mediated by cortico-subcortical circuits also involved in eye movements and attentional orienting. As such, tDCS could be applied to brain areas and networks beyond the occipital cortex, such as the temporo-parietal cortex, an important hub for visuo-spatial functions and a heteromodal region for visual and auditory processing [e.g., (38)]. For instance, we have investigated the effects of parietal neuromodulation on visuo-spatial orienting and visual search in multisensory contexts such as those used to rehabilitate HVFDs (39, 40). Notably, in the study of Bolognini et al. (40) on healthy individuals, the authors found that right anodal parietal tDCS (compared to sham or left parietal tDCS) increased the effects of a multisensory visual search training and these effects transferred to non-trained tasks of visual orienting and visuo-spatial attention. These results open the way to parietal tDCS to increase visuo-spatial functions also in HVFDs patients undergoing multisensory trainings.

Thus, in the present proof-of-principle study on brain-damaged patients with chronic HVFDs, we investigate the behavioral effects of a single application of anodal tDCS over the ipsilesional occipital cortex, the ipsilesional or the contralesional posterior parietal cortex (PPC), combined with multisensory detection task. The aims are multiple: first, the comparison of an occipital tDCS protocol (previously combined with restitutive approaches) with a parietal tDCS protocol (40) not yet tested in individuals with HVFDs. Moreover, in post-stroke rehabilitation NIBS often targets interhemispheric (im)balance, with the aim of reducing the hyperactivation of the spared, contralesional, hemisphere or of increasing the excitation of the ipsilesional one [e.g., (24, 37, 41)]. Nonetheless, a more complex model taking into account the amount of lesional load (42) highlights the central role of the spared hemisphere – whose plasticity should be promoted with excitatory protocols – when the ipsilesional one has sustained large structural damages. Accordingly, we also investigated the effects of contralesional parietal tDCS, characterizing the extension and location of grey and white matter, by means of lesion mapping. Finally, we explored clinical, lesional, or behavioral differences between left and right brain-damaged participants.

## 2 Materials and methods

### 2.1 Participants

The sample size was calculated *a priori* with G*Power (3.1.9.6; Heinrich-Heine-Universität Düsseldorf, Germany) considering: beta = 0.08, alpha = 0.05, a medium-small effect size [*f* = 0.20; (43, 44)], 8 measurements (i.e., 4 tDCS sessions x 2 Timepoints; see below), a strong correlation between repeated measures of 0.7, and sphericity correction = 1. The analysis indicated a minimum sample size of 15.

Accordingly, 15 brain-damaged individuals (mean age = 53 ± 15y; range 26-75y) with chronic HVFDs (median disease duration duration = 258 days, IQR = 194 days; range 154–3,272 days) were recruited at the Neuro-Ophthalmology Center and Ocular Electrophysiology Laboratory of IRCSS Istituto Auxologico Italiano hospital (Milan, Italy). Nine patients presented with right HVFDs, whereas six patients had left HVFDs, as a result of ischemic/hemorrhagic stroke (*N* = 13) or traumatic brain injury (TBI; *N* = 2). Clinical and demographic feature of the patients are reported in Table 1. All patients had no hearing deficits and did not present counterindications to tDCS (45). Finally, no participant showed signs of hemispatial neglect, assessed with a visuo-spatial battery including: line bisection (46), copy of Gainotti’s figure (47), letter cancelation (48), star cancelation (49), and bell cancelation (50).

**Table 1:** Demographic and clinical data.

Patient	Age/Sex	Disease duration (days)	Lesion etiology	Lesion site	HVFD
P1	52/M	181	I	Left, temporo-occipital	Right incomplete HH
P2	43/M	154	I	Left, nucleo-basal	Right incomplete HH
P3	66/M	400	I	Left, temporo-occipital	Right superior scotoma
P4	57/M	239	I	Left, mesial occipital	Right incomplete HH
P5	67/M	339	I	Left, temporo-occipital	Right superior quadrantopia
P6	45/M	258	I	Left, temporo-occipito-parietal	Right incomplete HH
P7	26/M	3,272	TBI	Left, temporal, hippocampal, frontal	Right inferior quadrantopia
P8	73/F	387	H	Left, parieto-occipital	Right incomplete HH
P9	53/M	258	TBI	Right, axonal damage	Left HH
P10	75/M	168	I	Right, occipital, polar and mesial	Left incomplete HH
P11	64/F	181	H	Right, parieto-occipital	Left HH
P12	57/M	180	I	Right, talamo-insular and medial temporal	Left superior quadrantopia
P13	31/F	297	H	Right, posterior	Left HH
P14	54/F	751	I	Right, vertebro-basilar event	Left incomplete HH
P15	55/F	110	I	Left, temporo-occipital	Right superior quadrantopia

I, ischemic stroke; H, hemorrhagic stroke; TBI, traumatic brain injury; HH, homonymous hemianopia.

The study was approved by the hospital Ethics Committee (Protocol ID: 25C901_2009) and was conducted in accordance to principles of the Declaration of Helsinki. All patients provided their written informed consent.

### 2.2 Lesion mapping

**Figure 1:**
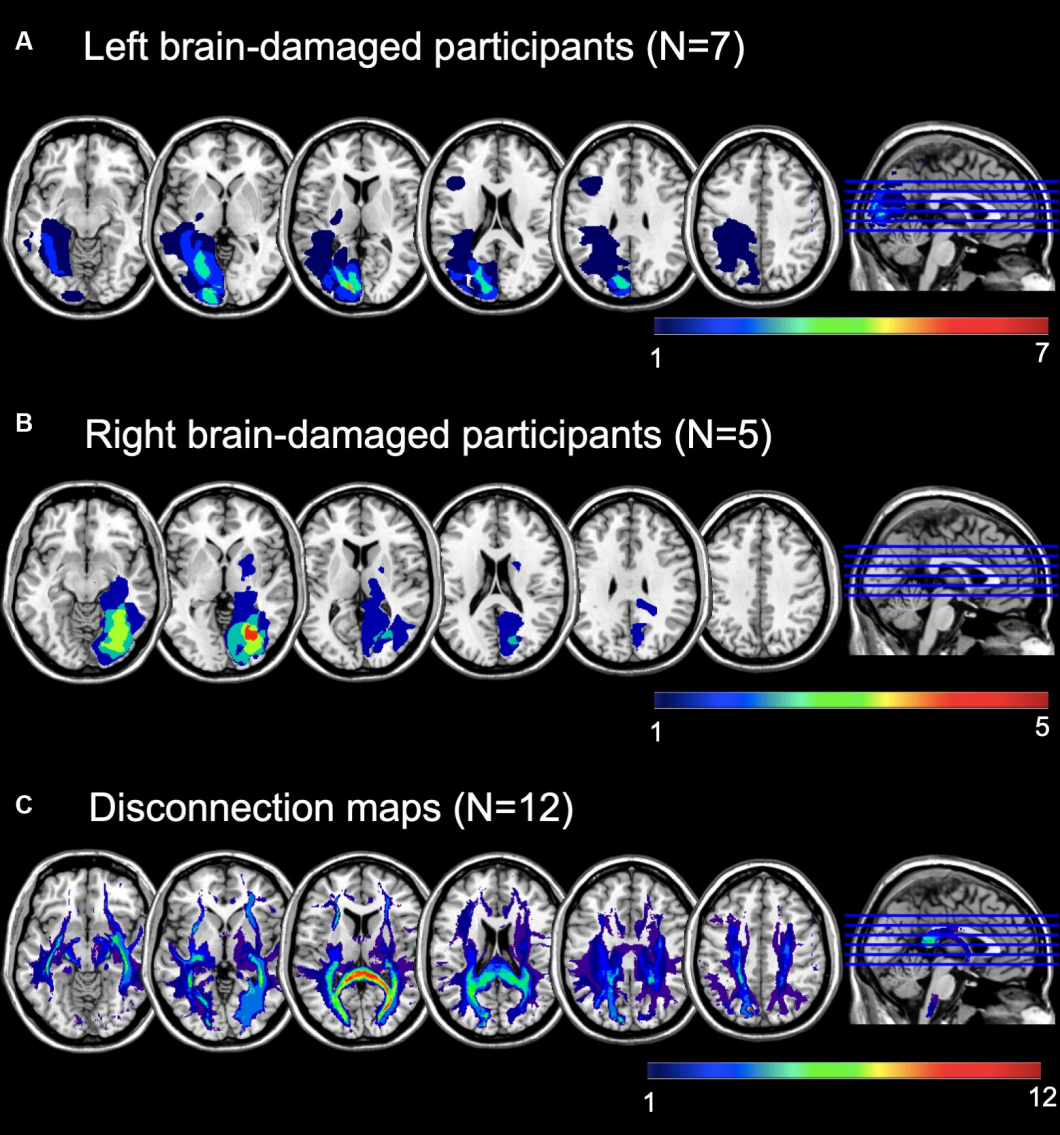
Lesion maps overlay of **(A)** left brain-damaged and **(B)** right brain-damaged participants with homonymous visual field defects (HVFDs). **(C)** Overlay of probabilistic disconnection maps of all participants. The lesion mapping was performed on the brain scans of 12 participants.

Brain imaging was available for *N* = 12. Brain lesions were delineated from Magnetic Resonance Imaging (MRI) scans in MRIcroGL (51). Brain scans and the lesion maps were normalized (function “MR segment-normalize”) onto an age-appropriate template by means of the Clinical Toolbox (52) for Statistical Parametric Mapping [SPM12, (53)] in MATLAB 2019b (54). Lesion maps overlays are depicted in Figure 1.

The mean lesion volume was 32.05 ± 36.54 cm3 (range = 0.24-127 cm3). According to the Automated Anatomical Labeling atlas [AAL, (55)], the most affected areas, irrespective of lesion side, were: the calcarine sulcus (*N* = 10), the lingual gyrus (*N* = 10), the superior (*N* = 8), the middle (*N* = 8), and the inferior (*N* = 7), occipital lobes, as well as the cuneus (*N* = 6), and the fusiform gyrus (*N* = 7). Moreover, we calculated the overall lesion extension (in number of voxels) of the occipital, temporal, and parietal lobes by summing the lesion extension of single areas. In more details, the occipital area included the calcarine sulcus, the cuneus, the lingual gyrus, the superior, middle, and inferior occipital gyrus; the temporal lobe included the inferior, middle, and superior gyri, as well as the fusiform area; for the parietal lobe, we included the post-central gyrus, the inferior and superior parietal lobules, the supramarginal gyrus, the angular gyrus, and the precunues. Lastly, because only few participants had temporal or parietal lesions, but they represent core areas of multisensory processing, attentional orienting and exploration abilities, we also calculated a temporo-parietal lesion burden, as the sum of lesion extension of parietal and temporal areas, excluding the more ventral fusiform gyrus. See also Table 2.

**Table 2:** Lesion extension of the injured lobes, gyri, and tracts.

Lobe/area/tract	Name	*N*	Lesion extension		Range
			Mean	SD	
Lobe	Occipital	10	13,901	10,669	0–30,540
	Parietal	6	3,520	10,287	0–35,896
	Temporal	8	5,383	6,929	0–17,303
	Temporo-parietal	8	5,756	14,057	0–49,584
Gyrus	Calcarine	10	0.24	0.22	0–0.71
	Cuneus	6	0.26	0.21	0–0.52
	Lingual	10	0.27	0.28	0–0.70
	Occipital Sup	8	0.16	0.21	0–0.53
	Occipital Mid	8	0.14	0.15	0–0.49
	Occipital Inf	7	0.33	0.30	0–0.73
	Fusiform	7	0.27	0.26	0–0.72
Tract	Cingulum	7	0.04	0.06	0–0.21
	Cingulum Ant	6	0.04	0.06	0–0.22
	Cingulum Post	7	0.09	0.15	0–0.40
	Corpus callosum	12	0.03	0.04	0–0.11
	Fronto Striatal	6	0.01	0.02	0–0.07
	IFOF	11	0.08	0.08	0–0.26
	ILF	10	0.10	0.10	0–0.27
	Optic Radiations	11	0.14	0.14	0–0.45
	Pons	6	0.02	0.03	0–0.08
	SLF II	6	0.05	0.12	0–0.42
	SLF III	6	0.05	0.10	0–0.34

Lesion extension of lobes are expressed as number of voxels. For gyri and tracts, values represent the proportion (0 to 1) of a lesioned area or tract. Sup, superior; Mid, middle; Inf, inferior; Ant, anterior; Post, posterior; IFOF, Inferior Fronto-Occipital Fasciculus; ILF, Inferior longitudinal fasciculus; SLF II and III, second and third branch of the superior longitudinal fasciculus.

Following a second co-registration on an MNI-152 template (slices thickness = 1 mm), the lesion maps were analyzed with Tractotron in Brain Connectivity Behaviour Toolkit^1^ (56) to obtain a probabilistic damage quantification of major white matter fibers. Lesion extension (i.e., the proportion of tract affected by the lesion) were considered of those tracts with a lesion probability >50%. As shown in Table 2 and Figure 1C, a number of intra- and inter-hemispheric white matter bundles were affected.

### 2.3 Audio-visual training

All participants underwent four different tDCS sessions (see the section “tDCS protocols”) during an audio-visual training [see (39)]. Specifically, participants were seated in front of a 2 m × 2 m training board (E.M.S. srl, Bologna, Italy, http://www.emsmedical.net), at a distance of 1.2 m, in a dimly lit room. The board featured 48 red light-emitting diodes (LED, diameter 1 cm, luminance 90 cd m2), distributed in six horizontal rows (eight lights per row). Forty-eight piezoelectric loudspeakers (0.4 W, 8 Ω) were located above each light, producing a white-noise (80 dB, duration 100 ms). Spatio-temporally congruent, cross-modal, audio-visual stimuli were presented at one out of 48 possible positions on the board. Participants were instructed to look at the fixation point – a yellow star (2°) located in the center of the apparatus – and to move their eyes to detect the presence of the visual stimulus (duration = 100 ms) by pressing right button of a wireless mouse. The inter-stimulus interval varied randomly between 1 and 3 s. False positive responses were recorded. The execution of oculomotor exploration by each participant was monitored visually by the experimenter, standing near the apparatus. The training included four blocks of 96 audio-visual trials (two trials for each spatial location), each lasting about 7 min, separated by small breaks. The duration of the training session was about 30 min. The task was programmed and run with E-Prime 2.0 (Psychology Software Tools, Pittsburgh, PA).

### 2.4 tDCS protocol

tDCS was delivered by a battery-driven current stimulator (BrainStim, E.M.S. srl, Bologna, Italy, http://www.emsmedical.net), through a couple of rubber-conductive electrodes inserted into saline-soaked sponges (anode/target electrode: 5 × 5 cm^2^ and cathode/reference electrode: 5 × 5 cm^2^). Anodal stimulation was applied to the target areas (see below) at 2 mA for 30 min, i.e., for the whole duration of the audio-visual detection task (40). In the case of sham tDCS, the stimulator was turned off after 30 s. The protocol was single-blind: whereas participants were kept blind to the tDCS condition (i.e., real or sham), the experimenters were not.

**Figure 2:**
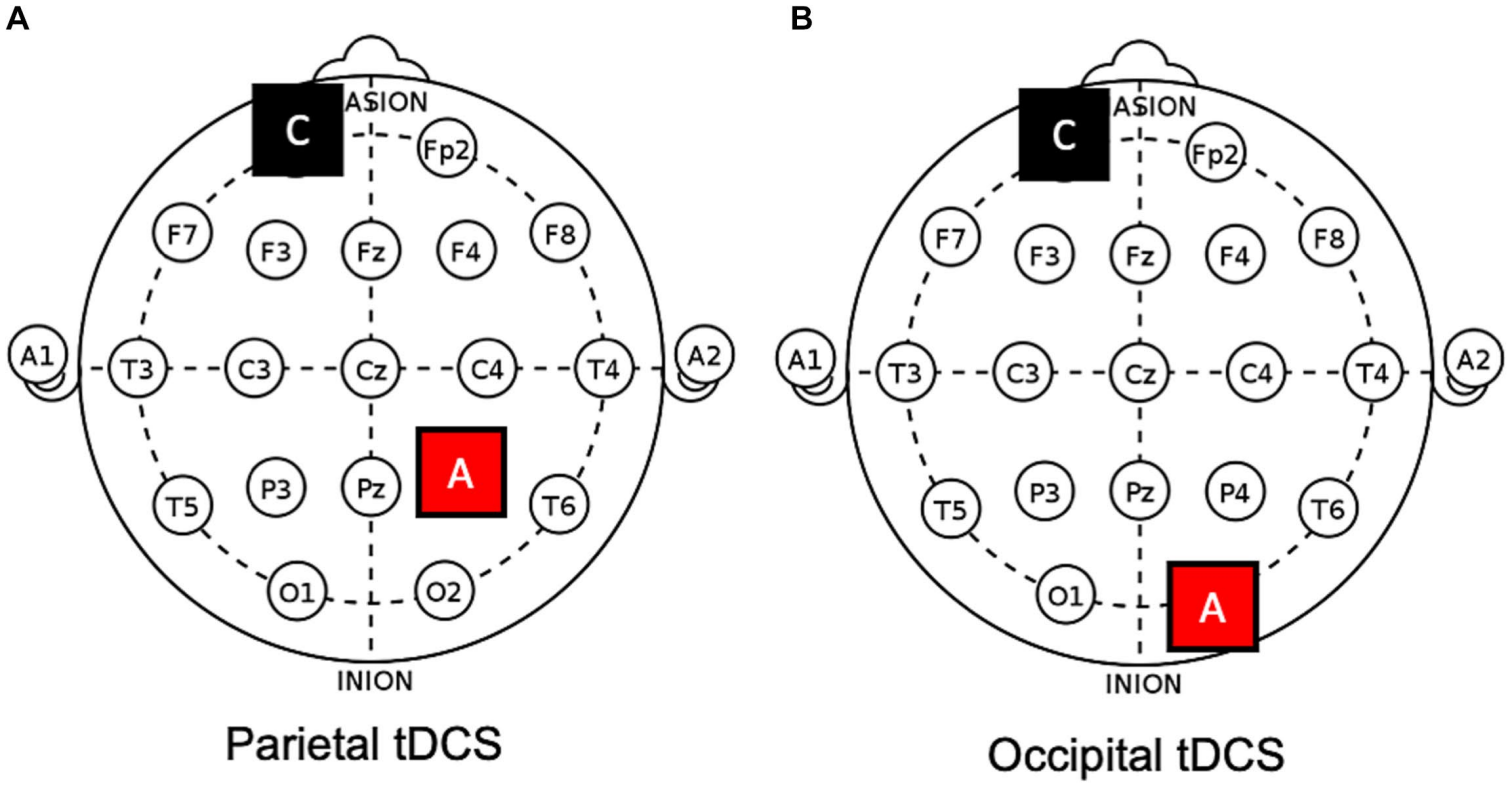
Electrodes positions on a 10–20 template. Examples of **(A)** parietal and **(B)** occipital anodal tDCS applied to the right hemisphere. A, anode; C, cathode.

On different days, four different protocols were applied in counterbalanced order, namely, (1) anodal tDCS over the ipsilesional occipital cortex (i.e., O1/O2 of the 10–20 EEG system; see also Figure 2), (2) anodal tDCS over the ipsilesional or (3) contralesional posterior parietal cortex (i.e., P3/P4), and (4) a sham protocol (i.e., the anode was positioned over one of the previous targets in a counterbalanced manner). The cathode was always placed in a supraorbital position (contralateral), contralateral to the anode. Sessions were separated by a wash-out period of, at least, one week.

No participants reported serious adverse effects. The most reported sensation was tingling under the anode at the beginning of the stimulation.

### 2.5 Assessment of the effects of the tDCS combined with the audio-visual training

The effects of audio-visual training session combined with tDCS were assessed by means of a visual detection task and two visual search tests, administered immediately before (t0) and at the end (t1) of the audio-visual detection task.

#### 2.5.1 Visual detection task with eye movements

By means of the multisensory panel used during the training, we measured unisensory visual detection abilities in the upper and lower parts of both the blind and the intact visual hemifields. Specifically, a visual target was presented for 100 ms in one of 48 different spatial positions (12 spatial locations in each quadrant of the visual field). For each of the 48 spatial positions, two trials were given, for a total of 24 trials in each of the four quadrants. In 24 catch trials no visual stimulus was given, in order to assess false positive responses. Participants were asked to press the button of a wireless mouse whenever they detected the visual stimulus. Eye movements were allowed, but after each trial the patient had to return to the fixation point; the next trial was presented only if the patients looked at the fixation. The total number of trials was 120 (96 with a target stimulus, and 24 catch trials). The number of detected targets and the corresponding response times (RTs) were registered.

#### 2.5.2 Visual search tests

Two different tasks were used to evaluate the participants’ ability to scan the visual field, namely, the EF and the Triangles tests. Both visual search paradigms were projected onto a wall (SONY-VPL-ES4 Projector); the stimulus arrays were displayed at a distance of 1.2 m from the participants’ eyes and subtended 35° × 28° of visual angle. Stimuli and responses were generated and measured by E-Prime 2 software. In both tasks, each trial began with a fixation (red cross, 1 s), which was followed by the presentation of the search array; participants were required to scan the visual field, searching for visual targets embedded among distracters (targets and distracters had the same size). No time limits were given, but participants were instructed to respond as accurately and as faster as possible. Following a response, a blank screen was presented for 1 s prior to the start of the next trial.

In the EF test, each array included 21 stimuli (all 5 cm x 5 cm), randomly distributed. The stimuli were green letters on a black background. Participants were instructed to search a single target (i.e., the green letter “F”) intermingled among distracters (i.e., the green letters “E”) and report the presence of the target with a keyboard: leftward arrow key if the target was present, or the rightward arrow key if absent. Twenty trials were presented: 16 trials in which the target was present, and four in which the target was absent. Correct responses and associated RTs were recorded. False alarms, namely the erroneous report of a target in an array where it was absent, were also recorded.

In the Triangles test, each array included 21 stimuli, randomly distributed on a black background. Different shapes of the same size (4 cm × 4 cm) were used as stimuli: yellow triangles as targets and yellow squares as distracters. The number of targets presented in the stimulus array ranged from 0 to 13 (Target/Distracters Ratio: 0–60%). As the number of targets increased, the number of distracters decreased, so that each stimulus array always included 21 stimuli. Participants were asked to scan the visual array and search for the total number of targets, pressing the space bar of the keyboard when they had finished to inspect the stimulus array. Immediately afterwards, they were asked to report verbally the number of detected targets. Twenty trials were given. For the Triangles test, too, we collected the amount of correct responses and RTs.

### 2.6 Analyses

All analyses were carried out with jamovi 2.3 [The jamovi project, (57)]. One participant (P15, see Table 1) dropped out after the first tDCS session because of personal reasons, thus the analyses were carried out on 14 participants.

For each tasks and for each participant, we calculated median RTs and accuracy (ACC), the last computed as the proportion of correct trials divided by the total number of trials (range: 0–1). For the unisensory visual detection task, RTs and ACC were calculated separately both for the blind and the sighted hemifields.

Because of strongly right-skewed distributions, RTs were analyzed by means of generalized mixed-models (GMM), fitting a gamma distribution (58, 59). Fixed effects were tested for “tDCS” (i.e., the 4 tDCS sessions), “Timepoint” (i.e., t0-pre and t1-post), and their interaction; random intercepts were calculated for participants. GMM of the unisensory visual detection task also included the factor “Hemifield” (i.e., blind and sighted). The distribution of ACC, on the other end, was strongly left-skewed (reflecting many high values). Therefore, ACC was transformed (1.1 – ACC) to obtain a right-skewed distribution with no 0 values, that was analyzed with the same procedures as for RTs. The significance of the fixed effects was evaluated by means of F-tests with the Satterthwaite method. Significant interactions were explored with Bonferroni-corrected post-hocs.

Moreover, we investigated differences between tDCS responders and non-responders with respect to baseline performance, clinical-demographic and lesion factors. Specifically, performance changes were calculated by subtracting the baseline (t0) performance from the post-intervention performance (i.e., Δpost-pre = post tDCS minus pre tDCS). For each task and tDCS session, participants were divided into two groups through a median split: tDCS responders (participants who showed larger improvements in Δpost-pre changes) and non-responders (participants who showed small or no improvements in Δpost-pre changes). Non-parametric comparisons between responders and non-responders (Mann–Whitney) were run for baseline performance, age, disease duration, lesion volume and lesion extension of the most involved brain areas and white-matter tracts.

Finally, we conducted explorative analysis considering the side of lesion. Specifically, Mann–Whitney tests were run to analyze possible differences between left and right brain-damaged participants in: age, disease duration, overall lesion volume and lesion extension of the occipital lesions. Moreover, for each behavioral task, left–right differences were calculated for baseline performance (i.e., the average performance at t0, before tDCS, across sessions), as well as tDCS-specific post-pre changes in ACC and RTs.

Data of the present work are available at: 10.5281/zenodo.10041851. This set of raw data is accessible under request because it includes sensitive information. Please, write your request to the corresponding author.

## 3 Results

### 3.1 Visual detection task

**Figure 3:**
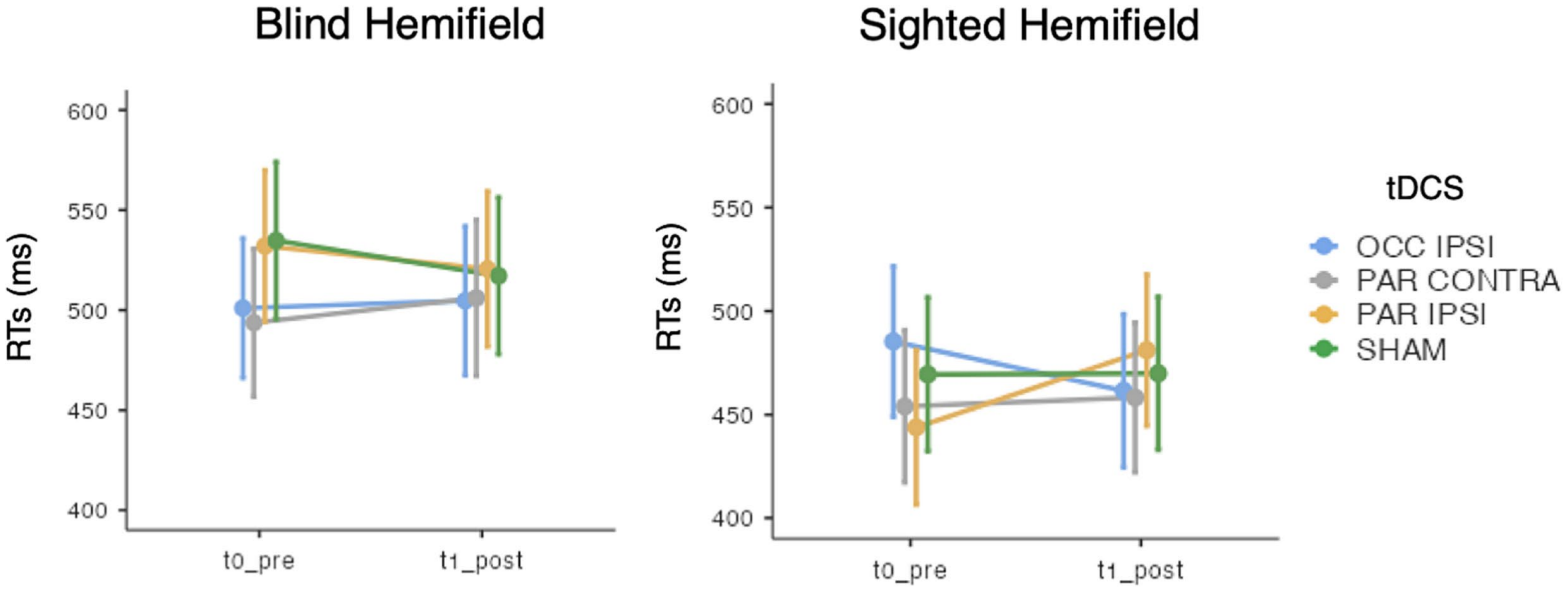
Mean response times (RTs) of the visual detection task, for each timepoint (t0_pre: baseline, t1_post: post-training), tDCS session (OCC IPSI: ipsilesional occipital tDCS; PAR IPSI: ipsilesional parietal tDCS; PAR CONTRA: contralesional parietal tDCS) and visual hemifield (blind vs. sighted). Error bars depict the SE.

In terms of ACC, the GMM indicated only an effect of Hemifield [χ^2^(1) = 295.84, *p* < 0.001], with higher accuracy rates in the spared visual hemifield (median = 0.96, interquantile range, IQR = 0.09) compared to the blind hemifield (median = 0.55, IQR = 0.5; see Table 3). No other significant main effects or interactions were found (lowest *p* = 0.569). As for the RTs, we observed an effect of Hemifield [*χ*^2^(1) = 295.84, *p* < 0.001; i.e., faster RTs in the spared hemifield] and a triple “Timepoint” x “tDCS” X “Hemifield” interaction [*χ*^2^(3) = 14.38, *p* = 0.002] whose post-hoc decomposition did not highlight significant post-tDCS effects for either hemifields (see Figure 3). Other effects did not reach the significance level (lowest *p* = 0.212). Detailed results are reported in the Supplementary material.

**Table 3:** Results of visual detection and visual search tasks.

tDCS	Task	Timepoint	ACC Mdn	ACC IQR	RTs Mdn	RTs IQR
Occ Ipsi	Visual Det – Blind	t0_pre	0.64	0.49	389	95
		t1_post	0.59	0.41	388	126
	Visual Det – Sighted	t0_pre	1.00	0.13	359	100
		t1_post	0.95	0.14	353	169
	EF	t0_pre	0.96	0.15	4,117	3,423
		t1_post	0.96	0.13	3,854	1,421
	Triangles	t0_pre	0.72	0.33	6,787	2,801
		t1_post	0.72	0.25	6,355	1940
Par Contra	Visual Det – Blind	t0_pre	0.55	0.27	391	220
		t1_post	0.55	0.48	373	188
	Visual Det – Sighted	t0_pre	1.00	0.09	312	109
		t1_post	0.95	0.13	331	97
	EF	t0_pre	0.96	0.11	3,850	1,601
		t1_post	0.92	0.09	3,480	1,673
	Triangles	t0_pre	0.67	0.22	5,904	1,528
		t1_post	0.78	0.25	5,916	1,454
Par Ipsi	Visual Det – Blind	t0_pre	0.59	0.55	404	234
		t1_post	0.52	0.44	400	177
	Visual Det – Sighted	t0_pre	1.00	0.08	321	182
		t1_post	0.95	0.13	374	184
	EF	t0_pre	0.92	0.16	3,777	1,174
		t1_post	0.96	0.13	3,253	1,202
	Triangles	t0_pre	0.72	0.25	6,122	1890
		t1_post	0.78	0.28	5,712	2,139
Sham	Visual Det – Blind	t0_pre	0.59	0.40	439	318
		t1_post	0.48	0.40	435	248
	Visual Det – Sighted	t0_pre	0.96	0.09	331	241
		t1_post	0.95	0.17	365	175
	EF	t0_pre	0.92	0.08	3,682	1736
		t1_post	0.96	0.08	3,635	1,559
	Triangles	t0_pre	0.72	0.30	6,926	2,949
		t1_post	0.83	0.14	6,598	1999

Median (Mdn) values and Inter quartile ranges (IQR) are reported for each behavioral task, tDCS protocol, and timepoint. Occ ipsi, ipsilesional occipital tDCS; Par contra, contralesional parietal tDCS; Par ipsi, ipsilesional parietal tDCS; Visual Det, visual detection task; Blind, blind visual hemifield; Sighted, sighted visual hemifield; ACC, accuracy; RTs, response times; Mdn, median; Occ, occipital; Par, parietal.

### 3.2 EF test

**Figure 4:**
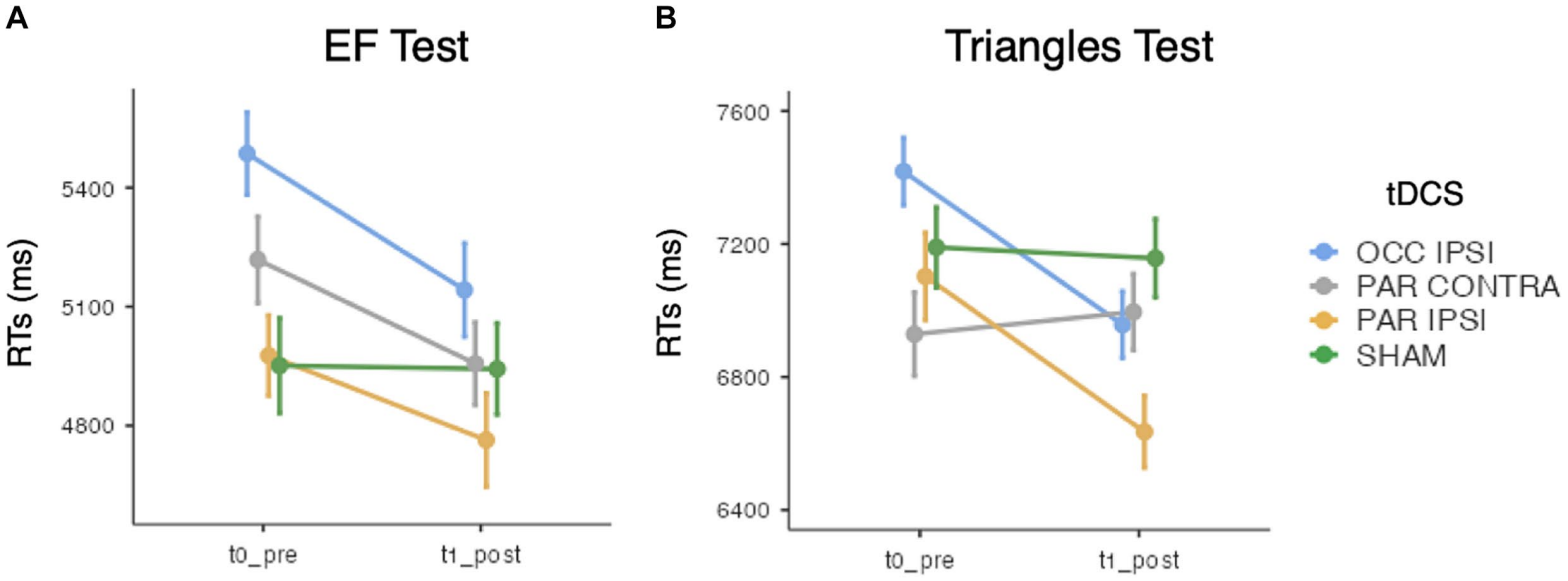
Mean search times (RTs) of **(A)** the EF test and **(B)** the Triangles test, for each timepoint and tDCS session. Error bars depict the SE. OCC, occipital tDCS; PAR, parietal tDCS; IPSI, ipsilesional; CONTRA, contralesional.

While ACC analysis showed no significant effects of tDCS session [*χ*^2^(3) = 0.15, *p* = 0.985], Timepoint [χ^2^(1) = 0.87, *p* = 0.352], or their interaction [*χ*^2^(3) = 1.24, *p* = 0.742], GMMs on RTs revealed significant effects of tDCS session [*χ*^2^(3) = 52.4, *p* < 0.001], Timepoint [*χ*^2^(1) = 11.5, *p* < 0.001], and their interaction [*χ*^2^(3) = 13.5, *p* < 0.001]. Specifically, a significant improvement was observed after ipsilesional occipital tDCS (pre-post difference = 344.2 ms ± 73.2 SE; z = 4.67; *p* < 0.001) but not after ipsilesional parietal (*p* = 0.495), contralesional parietal (*p* = 0.09) or sham tDCS (*p* = 1). See Figure 4A.

### 3.3 Triangles test

Triangles ACC analysis did not show main effects of tDCS session [χ^2^(3) = 0.78, *p* = 0.854] and Timepoint [χ^2^(1) = 2, *p* = 0.157], nor their interaction [χ^2^(3) = 0.89, *p* = 0.831]. Instead, with respect to RTs, the effect of tDCS session [*χ*^2^(3) = 24.1, *p* < 0.001], Timepoint [*χ*^2^(1) = 10.4, *p* = 0.001], and their interaction [*χ*^2^(3) = 75, *p* < 0.001] reached the significance level. Of relevance, RTs were faster after ipsilesional occipital (pre-post difference = 460.9 ms ± 77.8 SE; *z* = 5.92; *p* < 0.001) and ipsilesional parietal tDCS (pre-post difference = 467.4 ms ± 112.1 SE; *z* = 4.15; *p* < 0.001). No changes were observed after contralesional parietal (pre-post difference = 66.5 ms ± 95.7 SE; *z* = −0.7; *p* = 1) or sham tDCS (pre-post difference = 32.5 ms ± 102.8 SE; *z* = 0.13; *p* = 1) (see Figure 4B).

### 3.4 Difference between tDCS responders and non-responders

In light of the previous results, responders and non-responders were categorized based on the RTs pre-post changes at the visual search tasks.

Regarding the EF test, we did not observe significant differences between tDCS responders and non-responders in baseline performance or in any clinical-demographic and lesional data (i.e., structural imaging), for ipsilesional occipital (lowest *u* = 8.5; lowest *p* = 0.124) or ipsilesional parietal tDCS (lowest *u* = 9; lowest *p* = 0.158). Nonetheless, responders to contralesional parietal tDCS presented with larger lesions of the IFOF (*U* = 5; *p* = 0.041) and of the temporal cortex (*U* = 5.5; *p* = 0.05). No other significant differences emerged (lowest *u* = 6; lowest *p* = 0.065).

Instead, responders to ipsilesional occipital tDCS at the Triangles test showed smaller lesions in the parietal cortex (*U* = 4.5; *p* = 0.026), as wells as in the first (*U* = 4.5; *p* = 0.026) and the second branch (*U* = 2.5; *p* = 0.017) of the SLF. Moreover, occipital tDCS responders had a shorter disease duration (*U* = 3.5; *p* = 0.009). No significant difference emerged between tDCS-responders and non-responders for ipsilesional parietal tDCS (lowest *u* = 10.5; lowest *p* = 0.254) or contralesional parietal tDCS (lowest *u* = 8; lowest *p* = 0.104).

### 3.5 Differences between left and right brain-damaged participants

Mann–Whitney tests revealed no significant differences between left and right brain-damaged participants in any demographical, clinical, or lesional variable (lowest *U* = 7, lowest *p* = 0.106).

With respect to the visual detection task, at baseline, no differences in accuracy emerged for the sighted (*U* = 20.5, *p* = 0.697) and the blind hemifield (*U* = 12, *p* = 0.142); with respect to RTs, right brain-damaged participants were slower (*U* = 6, *p* = 0.02; 584 ± 200 ms) than left brain-damaged ones (355 ± 64.7 ms) at detecting visual stimuli in the blind hemifield, but not in the sighted one (*U* = 11, *p* = 0.108). No significant differences emerged for any tDCS protocol or hemifield in terms of ACC (lowest *U* = 15.5, lowest *p* = 0.301) or RTs (lowest *U* = 10, lowest *p* = 0.081).

Regarding the EF test, no significant differences emerged with respect to the baseline performance (ACC: *U* = 15, *p* = 0.270; RTs: *U* = 18, *p* = 0.491) and tDCS effects on ACC (Δpost-pre; lowest *U* = 17, lowest *p* = 0.388) for any tDCS session. The only significant difference was related to RTs change after ipsilesional parietal tDCS (Δpost-pre = 921 ± 837 ms, *U* = 2, *p* = 0.003), with a greater RTs reduction in participants with right-sided hemispheric lesions as compared to left-sided ones (332 ± 505 ms).

Similarly, the analysis of Triangles test performance did not reveal differences in ACC (*U* = 15, *p* = 0.282) and RTs (*U* = 20, *p* = 0.662) at the baseline, nor ACC differences for any tDCS stimulation (lowest *U* = 17, lowest *p* = 0.388). Instead, a larger RTs reduction after occipital tDCS emerged for right brain-damaged participants (1,027 ± 735 ms; vs. left brain-damaged participants: 47.7 ± 711).

## 4 Discussion

In the present study, we investigated the behavioral effects of a single application of anodal occipital and parietal tDCS applied during a multisensory audio-visual training aimed at promoting compensatory oculomotor scanning behavior in brain-damaged patients with chronic HVFDs. To our knowledge, this is the first time that tDCS has been combined with a compensatory training for chronic HVFDs. In previous clinical reports, occipital tDCS was combined with multiple-session restorative trainings [e.g., (35)]. Here, instead, we performed an explorative study to assess the potential benefit of combining tDCS during an audio-visual detection training involving multisensory and attentional orienting networks.

Overall, we found that both ipsilesional occipital and ipsilesional parietal anodal tDCS accelerated visual field scanning (EF and Triangle tests) immediately at the end of a single 30-min training, whereas sham tDCS did not improve the performance of brain-damaged patients in a chronic stage of illness.

Early tDCS studies [e.g., Antal et al. (60, 61)] demonstrated that the application of offline tDCS on primary visual areas can induce polarity-dependent, short-lived, changes in the excitability of the underlying cortex; in particular, anodal tDCS was associated with an increase in excitability of the primary visual cortex, as indexed by a transient reduction of TMS-induced phosphenes threshold [Antal et al. (60)], and an increase in the N70 component of visual evoked potentials (61). Accordingly, occipital tDCS has been combined with restorative trainings (21, 22, 34), to boost the excitability of the underlying, hypofunctioning, visual areas in synergy with the rationale of visual restitution therapies. In our investigation, however, tDCS was applied on-line during a multisensory training requiring participants to overtly orient their attention toward audio-visual stimuli. Therefore, considering that the effects of occipital tDCS were observed in visual search tasks and that the effects of on-line protocols are known modulate task-related networks [e.g., (62)], we argue that the effects of occipital anodal stimulation go beyond the excitation of the underlying primary visual areas, possibly modulating those circuits mediating eye movements, including cortical–subcortical structures of the extra-geniculate pathways projecting to temporo-parietal areas, part of multisensory circuits and crucial for the deployment of visuospatial attention.

In this regard, we also found that responders to occipital tDCS in the Triangle test had smaller lesions affecting the parietal cortex and two branches of the Superior Longitudinal Fasciculus, a white matter tract connecting posterior and frontal areas that plays a main role in attentional orienting [Corbetta and Shulman, (63, 64)]. With respect to this, Bueichekù et al. (65) demonstrated that the strength of functional connectivity (even “at rest”) between primary visual areas and the posterior parietal lobule is a predictor of visual search efficiency; thus, the neuromodulation of these functional networks may improve visuo-spatial abilities, in turn speeding up visual search performance after a posterior stroke.

Moreover, tDCS applied over the posterior parietal cortex, an important hub of the network for visuospatial attention [Corbetta and Shulmann, (63)], improved visual search in patients with chronic HVFDs. Indeed, a facilitatory effect on RTs emerged in the Triangles test after ipsilesional parietal stimulation, in line with previous evidence (40), which showed, in healthy participants, that anodal parietal tDCS over the right hemisphere enhanced the effects of an audiovisual training as indexed by decreased latencies in visual search tasks. Furthermore, the effects of parietal neuromodulation on visuospatial functions have been demonstrated by other works investigating attentional orienting [Sparing et al. (39, 43, 66, 67)].

The effects of parietal tDCS on attentional orienting and visuospatial attention should also be interpreted from the perspective of interhemispheric balance. Indeed, the (un)balanced excitability of intra- and interhemispheric circuits is the focus of NIBS interventions for the recovery of motor functions (68), language deficits (69) and HVFDs (37). Accordingly, increasing the excitability of the lesioned hemisphere or reducing the, presumably pathological excitation of the spared one would promote functional recovery. With regard to visuospatial attention, this model was shown to be effective in diminishing the attentional asymmetries of hemispatial neglect (70). Within this framework, the present findings suggest that even in chronic HVFDs, the increased excitability and strengthening of the affected parietal circuits brought about anodal parietal tDCS may favor a better deployment of attention to the contralateral, blind, hemifield, resulting in an overall improvement of the visual exploration of such contralesional sector of the space. Nonetheless, contralesional parietal tDCS seems to boost search times but selectively in those participants with larger lesions of the temporo-parietal cortex or of white matter tracts connecting the occipital cortex with temporal and frontal areas. These results are in line with the bimodal hypothesis (42, 71) of plasticity modulation in brain recovery: in those patients with larger brain lesions, the excitability of the contralesional hemisphere should be promoted (rather than inhibited) as it would compensate for functions previously mediated by the lesioned one. In our sample of chronic patients with larger lesions, contralesional tDCS may have boosted those circuits that adapted to brain lesions to subserve visual exploration.

Finally, in light of the well-known hemispheric differences in visuospatial processing, we further characterized the effects of ipsilesional and contralesional tDCS considering the possible role of the hemispheric side of the lesion. Overall, the explorative analyses showed that patients with right-sided hemispheric damage seemed to benefit more from the ipsilesional occipital (Triangles test) and parietal (EF test) tDCS than left-lesioned ones. These results depict a complex relationship between lesion side, functional lateralization, and inter-hemispheric balance asymmetries. As for parietal tDCS, hemispheric asymmetries of fronto-parietal attentional circuits have been largely investigated [Corbetta and Shulmann, (63)], with a certain specialization for the right hemisphere, especially, for a ventral circuit mediating attentional re-orienting. Moreover, Bolognini et al. (40) found that anodal tDCS of the right PPC improved visual search performance. Here, however, patients with a left-sided brain lesion did benefit from right parietal (i.e., contralesional) tDCS. Therefore, ipsilesional excitatory parietal stimulation seems to be more effective to reactivate core areas of right-sided injured parietal circuits. Although such inter-hemispheric asymmetries are well characterized for parietally-mediated visuospatial attention, evidence about structural or functional asymmetries in the occipital pathways of patients with HVFDs remains limited (37). A recent study by Pietrelli et al. (72) analyzed the oscillatory activity of the posterior cortices of hemianopic patients and found an imbalanced inter-hemispheric alpha activity, which could predict the performance in visuo-spatial tasks. Notably, patients with right-sided hemispheric lesions showed more alterations of the oscillatory activity and a stronger inter-hemispheric imbalance, possibly mediated by cortico-cortical connections of the more right-lateralized parietal networks. Overall, this intriguing account may explain the larger benefits brought about by ipsilesional occipital and parietal tDCS in the right-damaged participants in our study. However, this hypothesis needs further confirmation, given the small- and not completely balanced- sample tested in the present work. The complex picture emerging from these results highlights the need for an optimal characterization of intra and inter-hemispheric functional mechanisms in HVFD (e.g., by means of EEG or functional MRI), as a crucial step toward a more tailored and effective application of NIBS for visual rehabilitation (73). Moreover, the understanding of HVFDs through the lenses of interhemispheric, oscillatory-based models may pave the way for other tES protocols beyond tDCS, such as the multifocal, cross-frequency transcranial alternate current stimulation (tACS) approach proposed by Raffin et al. (2020), or tDCS-tACS integrated protocols (24, 37).

The results discussed so far are confined to a reduction of RTs in visual search; in fact, the ACC was not modulated by tDCS. This null result is not surprising: a single application of tDCS, as well as a single training session, cannot be sufficient to induce more robust changes at the level of accuracy in patients with chronic VFDs. In fact, conventional multisensory trainings require multiple days of training [usually 2–3 weeks; (17)], especially in chronic patients whose neuroplastic changes require longer training than in sub-acute ones (21). We believe the same reasoning applies to the null findings on the visual detection task where no gains in speed and accuracy were induced by the multisensory training, whether coupled with real or sham stimulation (40).

The present study has some limitations that need to be mentioned. First, we acknowledge that the RTs improvements discussed above are small; nonetheless, we believe such changes in visual search after a single tDCS session coupled with multisensory training represent a promising finding, considering that visual search by itself was not trained, suggesting a transfer of training that could be larger and stronger after multiple days of training. However, a future study should better characterize the impact of other factors that may affect behavioral performance and rehabilitation, such as the level of alertness of the participants. Finally, whereas the sample size appeared sufficient for repeated-measure analyses, it should be increased to observe reliable correlations with clinical and lesional variables and should be better balanced for the lesional side to draw solid conclusions about hemispheric asymmetries.

In conclusion, the present results suggest that occipital and parietal tDCS combined with an audiovisual training may improve visual search speed in HVFDs. Importantly, however, these effects have to be confirmed and extended by repeated sessions of tDCS combined with multisensory trainings, after which, we may expect faster behavioral improvement with a positive impact on the activities of daily living.

## Data availability statement

The datasets presented in this study can be found in online repositories. The names of the repository/repositories and accession number(s) can be found at DOI: 10.5281/zenodo.10041851.

## Ethics statement

The studies involving humans were approved by Ethics Committee of IRCCS Istituto Auxologico Italiano. The studies were conducted in accordance with the local legislation and institutional requirements. The participants provided their written informed consent to participate in this study.

## Author contributions

LD: Conceptualization, Data curation, Formal analysis, Methodology, Visualization, Writing – original draft, Writing – review & editing. CC: Conceptualization, Data curation, Investigation, Methodology, Project administration, Writing – review & editing. LM: Investigation, Methodology, Writing – review & editing. SBM: Investigation, Methodology, Writing – review & editing. NB: Conceptualization, Methodology, Project administration, Resources, Supervision, Writing – review & editing.

## Footnotes

^1^http://toolkit.bcblab.com/
