# Supplementary material for: The effects of occipital and parietal tDCS on chronic visual field defects after brain injury
Source: Front Neurol. 2024 Feb 14;15:1340365. doi: 10.3389/fneur.2024.1340365 (PMC10899507; doi:10.3389/fneur.2024.1340365)
Supplement: Supplementary file 1 [file Table_1.DOCX]

Supplementary Material

The Effects of Occipital and Parietal tDCS on Chronic Visual Field Defects after Brain Injury

# Visual detection Task

## Generalized Mixed Model – ACC

Results of the Generalized Mixed Model (GMM) on the accuracy (ACC) of the Visual Detection Task are reported in the Supplementary Tables 1, 2, and 3.

**Supplementary Table 1. Model Info**

| **Info** | **Value** | **Comment** |
| --- | --- | --- |
| Model Type | Custom | Model with custom family |
| Call | glm | ACC_INV ~ 1 + TDCS + Timepoint + Hemifield + TDCS:Timepoint + TDCS:Hemifield + Timepoint:Hemifield + TDCS:Timepoint:Hemifield + (1 \| ID) |
| Link function | Identity | Coefficients in the same scale of y |
| Distribution | Gamma | Skewed continuous distribution |
| LogLikel. | 249.871 | More is better |
| R-squared | NaN | Marginal |
| R-squared | NaN | Conditional |
| AIC | -463.740 | Less is better |
| BIC | -389.855 | Less is better |
| Deviance | 125.444 | Conditional |
| Residual DF | 430.000 |  |
| Chi-squared/DF | 0.279 | Overdispersion indicator |
| Converged | yes |  |
| Optimizer | bobyqa |  |

**Supplementary Table 2. Fixed Effect Omnibus tests**

| **Factor** | **X²** | **df** | **p** |
| --- | --- | --- | --- |
| TDCS | 1.836 | 3.00 | 0.607 |
| Timepoint | 0.227 | 1.00 | 0.634 |
| Hemifield | 295.843 | 1.00 | < .001 |
| TDCS ✻ Timepoint | 1.614 | 3.00 | 0.656 |
| TDCS ✻ Hemifield | 1.102 | 3.00 | 0.777 |
| Timepoint ✻ Hemifield | 0.325 | 1.00 | 0.569 |
| TDCS ✻ Timepoint ✻ Hemifield | 0.482 | 3.00 | 0.923 |

**Supplementary Table 3. Random Components**

| **Groups** | **Name** | **SD** | **Variance** | **ICC** |
| --- | --- | --- | --- | --- |
| ID | (Intercept) | 0.0434 | 0.00188 | 0.00188 |
| Residual |  | 0.5301 | 0.28099 |  |
| Residuals |  | 0.5301 | 0.28099 | . |

Note. Number of Obs: 448 , groups: ID 14

## Generalized Mixed Model – RTs

Results of the GMM on Response Times (RTs) of the Visual Detection Task are reported in the Supplementary Tables 4, 5, 6, and 7.

**Supplementary Table 4. Model Info**

| **Info** | **Value** | **Comment** |
| --- | --- | --- |
| Model Type | Custom | Model with custom family |
| Call | glm | RT ~ 1 + TDCS + Timepoint + Hemifield + TDCS:Timepoint + TDCS:Hemifield + Timepoint:Hemifield + TDCS:Timepoint:Hemifield + (1 \| ID) |
| Link function | Identity | Coefficients in the same scale of y |
| Distribution | Gamma | Skewed continuous distribution |
| LogLikel. | -2749.6474 | More is better |
| R-squared | NaN | Marginal |
| R-squared | NaN | Conditional |
| AIC | 5535.2900 | Less is better |
| BIC | 5609.1810 | Less is better |
| Deviance | 32.8858 | Conditional |
| Residual DF | 430.0000 |  |
| Chi-squared/DF | 0.0943 | Overdispersion indicator |
| Converged | yes |  |
| Optimizer | bobyqa |  |

**Supplementary Table 5. Fixed Effect Omnibus tests**

| **Factor** | **X²** | **df** | **p** |
| --- | --- | --- | --- |
| TDCS | 4.4991 | 3.00 | 0.212 |
| Timepoint | 0.0797 | 1.00 | 0.778 |
| Hemifield | 30.4586 | 1.00 | < .001 |
| TDCS ✻ Timepoint | 2.8184 | 3.00 | 0.420 |
| TDCS ✻ Hemifield | 4.3304 | 3.00 | 0.228 |
| Timepoint ✻ Hemifield | 0.5523 | 1.00 | 0.457 |
| TDCS ✻ Timepoint ✻ Hemifield | 14.3828 | 3.00 | 0.002 |

**Supplementary Table 6. Random Components**

| **Groups** | **Name** | **SD** | **Variance** | **ICC** |
| --- | --- | --- | --- | --- |
| ID | (Intercept) | 101.613 | 10325.2528 | 1.000 |
| Residual |  | 0.315 | 0.0995 |  |
| Residuals |  | 0.315 | 0.0995 | . |

Note. Number of Obs: 448 , groups: ID 14

**Supplementary Table 7. Post-hoc comparisons of the significant interaction “TDCS ✻ Timepoint ✻ Hemifield”**

| **TDCS** | **Hemifield** | **Comparison** | **Difference** | **SE** | **z** | **P (Bonferroni)** |
| --- | --- | --- | --- | --- | --- | --- |
| OCC IPSI | HEMI | t0 – t1 | -4.08 | 17.0 | -0.2407 | 1.000 |
| OCC IPSI | OK | t0 – t1 | 27.17 | 17.8 | 1.5299 | 1.000 |
| SHAM | HEMI | t0 – t1 | 11.77 | 22.8 | 0.5173 | 1.000 |
| SHAM | OK | t0 – t1 | -6.19 | 20.9 | -0.2970 | 1.000 |
| PAR IPSI | HEMI | t0 – t1 | 31.34 | 23.8 | 1.3165 | 1.000 |
| PAR IPSI | OK | t0 – t1 | -14.90 | 20.3 | -0.7354 | 1.000 |
| PAR CONTRA | HEMI | t0 – t1 | -13.69 | 23.3 | -0.5868 | 1.000 |
| PAR CONTRA | OK | t0 – t1 | -20.04 | 19.9 | -1.0082 | 1.000 |

Note. For the sake of readability, only pre-post comparisons within each hemifield and tDCS session are reported. OCC IPSI: ipsilesional occipital tDCS; PAR IPSI: ipsilesional parietal tDCS; PAR CONTRA: contralesional parietal tDCS.

# EF Task

## Generalized Mixed Model – ACC

Results of the GMM on ACC of the EF Task are reported in the Supplementary Tables 8, 9, and 10.

**Supplementary Table 8. Model Info**

| **Info** | **Value** | **Comment** |
| --- | --- | --- |
| Model Type | Custom | Model with custom family |
| Call | glm | ACC_INV ~ 1 + TDCS + Timepoint + TDCS:Timepoint + (1 \| ID) |
| Link function | Identity | Coefficients in the same scale of y |
| Distribution | Gamma | Skewed continuous distribution |
| LogLikel. | 175.3030 | More is better |
| R-squared | NaN | Marginal |
| R-squared | NaN | Conditional |
| AIC | -330.6100 | Less is better |
| BIC | -303.4209 | Less is better |
| Deviance | 7.6687 | Conditional |
| Residual DF | 102.0000 |  |
| Chi-squared/DF | 0.0736 | Overdispersion indicator |
| Converged | yes |  |
| Optimizer | bobyqa |  |

**Supplementary Table 9. Fixed Effect Omnibus tests**

| **Factor** | **X²** | **df** | **p** |
| --- | --- | --- | --- |
| TDCS | 0.152 | 3.00 | 0.985 |
| Timepoint | 0.865 | 1.00 | 0.352 |
| TDCS ✻ Timepoint | 1.244 | 3.00 | 0.742 |

**Supplementary Table 10. Random Components**

| **Groups** | **Name** | **SD** | **Variance** | **ICC** |
| --- | --- | --- | --- | --- |
| ID | (Intercept) | 0.0411 | 0.00169 | 0.00169 |
| Residual |  | 0.2933 | 0.08604 |  |
| Residuals |  | 0.2933 | 0.08604 | . |

Note. Number of Obs: 112 , groups: ID 14

## Generalized Mixed Model – RTs

Results of the GMM on RTs of the EF Task are reported in the Supplementary Tables 11, 12, 13, and 14.

**Supplementary Table 11. Model Info**

| **Info** | **Value** | **Comment** |
| --- | --- | --- |
| Model Type | Custom | Model with custom family |
| Call | glm | RT ~ 1 + TDCS + Timepoint + TDCS:Timepoint + (1 \| ID) |
| Link function | Identity | Coefficients in the same scale of y |
| Distribution | Gamma | Skewed continuous distribution |
| LogLikel. | -877.6917 | More is better |
| R-squared | NaN | Marginal |
| R-squared | NaN | Conditional |
| AIC | 1775.3800 | Less is better |
| BIC | 1802.5684 | Less is better |
| Deviance | 1.8222 | Conditional |
| Residual DF | 102.0000 |  |
| Chi-squared/DF | 0.0184 | Overdispersion indicator |
| Converged | yes |  |
| Optimizer | bobyqa |  |

**Supplementary Table 12. Fixed Effect Omnibus tests**

| **Factor** | **X²** | **df** | **p** |
| --- | --- | --- | --- |
| TDCS | 52.4 | 3.00 | < .001 |
| Timepoint | 11.5 | 1.00 | < .001 |
| TDCS ✻ Timepoint | 13.5 | 3.00 | 0.004 |

**Supplementary Table 13. Random Components**

| **Groups** | **Name** | **SD** | **Variance** | **ICC** |
| --- | --- | --- | --- | --- |
| ID | (Intercept) | 948.527 | 899703.2206 | 1.000 |
| Residual |  | 0.168 | 0.0283 |  |
| Residuals |  | 0.168 | 0.0283 | . |

Note. Number of Obs: 112 , groups: ID 14

**Supplementary Table 14. Post-hoc comparisons of the significant interaction “TDCS ✻ Timepoint”**

| **TDCS** | **Comparison** | **Difference** | **SE** | **z** | **P (Bonferroni)** |
| --- | --- | --- | --- | --- | --- |
| OCC IPSI | t0 – t1 | 344.21 | 73.9 | 4.66 | <0.001 |
| SHAM | t0 – t1 | 8.32 | 94.8 | 0.09 | 1.000 |
| PAR IPSI | t0 – t1 | 213.19 | 89.9 | 2.37 | 0.495 |
| PAR CONTRA | t0 – t1 | 261.99 | 88.8 | 2.95 | 0.089 |

Note. For the sake of readability, only pre-post comparisons within each tDCS session are reported. OCC IPSI: ipsilesional occipital tDCS; PAR IPSI: ipsilesional parietal tDCS; PAR CONTRA: contralesional parietal tDCS.

# Triangles Task

## Generalized Mixed Model – ACC

Results of the GMM on ACC of the Triangles Task are reported in the Supplementary Tables 15, 16, and 17.

**Supplementary Table 15. Model Info**

| **Info** | **Value** | **Comment** |
| --- | --- | --- |
| Model Type | Custom | Model with custom family |
| Call | glm | ACC_REV ~ 1 + TDCS + Timepoint + TDCS:Timepoint + (1 \| ID) |
| Link function | Identity | Coefficients in the same scale of y |
| Distribution | Gamma | Skewed continuous distribution |
| LogLikel. | 94.5708 | More is better |
| R-squared | NaN | Marginal |
| R-squared | NaN | Conditional |
| AIC | -169.1400 | Less is better |
| BIC | -141.9566 | Less is better |
| Deviance | 9.1838 | Conditional |
| Residual DF | 102.0000 |  |
| Chi-squared/DF | 0.0830 | Overdispersion indicator |
| Converged | yes |  |
| Optimizer | bobyqa |  |

**Supplementary Table 16. Fixed Effect Omnibus tests**

| **Factor** | **X²** | **df** | **p** |
| --- | --- | --- | --- |
| TDCS | 0.780 | 3.00 | 0.854 |
| Timepoint | 2.004 | 1.00 | 0.157 |
| TDCS ✻ Timepoint | 0.878 | 3.00 | 0.831 |

**Supplementary Table 17. Random Components**

| **Groups** | **Name** | **SD** | **Variance** | **ICC** |
| --- | --- | --- | --- | --- |
| ID | (Intercept) | 0.0885 | 0.00783 | 0.00777 |
| Residual |  | 0.3240 | 0.10496 |  |
| Residuals |  | 0.3240 | 0.10496 | . |

Note. Number of Obs: 112 , groups: ID 14

## Generalized Mixed Model – RTs

Results of the GMM on RTs of the Triangles Task are reported in the Supplementary Tables 18, 19, 20, and 21.

**Supplementary Table 18. Model Info**

| **Info** | **Value** | **Comment** |
| --- | --- | --- |
| Model Type | Custom | Model with custom family |
| Call | glm | RT ~ 1 + Timepoint + TDCS + Timepoint:TDCS + (1 \| ID) |
| Link function | Identity | Coefficients in the same scale of y |
| Distribution | Gamma | Skewed continuous distribution |
| LogLikel. | -908.4711 | More is better |
| R-squared | NaN | Marginal |
| R-squared | NaN | Conditional |
| AIC | 1836.9400 | Less is better |
| BIC | 1864.1271 | Less is better |
| Deviance | 1.3276 | Conditional |
| Residual DF | 102.0000 |  |
| Chi-squared/DF | 0.0140 | Overdispersion indicator |
| Converged | yes |  |
| Optimizer | bobyqa |  |

**Supplementary Table 19. Fixed Effect Omnibus tests**

| **Factor** | **X²** | **df** | **p** |
| --- | --- | --- | --- |
| Timepoint | 10.4 | 1.00 | 0.001 |
| TDCS | 24.1 | 3.00 | < .001 |
| Timepoint ✻ TDCS | 75.0 | 3.00 | < .001 |

**Supplementary Table 20. Random Components**

| **Groups** | **Name** | **SD** | **Variance** | **ICC** |
| --- | --- | --- | --- | --- |
| ID | (Intercept) | 1004.285 | 1.01e+6 | 1.000 |
| Residual |  | 0.137 | 0.0188 |  |
| Residuals |  | 0.137 | 0.0188 | . |

Note. Number of Obs: 112 , groups: ID 14

**Supplementary Table 21. Post-hoc comparisons of the significant interaction “TDCS ✻ Timepoint”**

| **TDCS** | **Comparison** | **Difference** | **SE** | **z** | **P (Bonferroni)** |
| --- | --- | --- | --- | --- | --- |
| OCC IPSI | t0 – t1 | 460.9 | 77.8 | 5.92 | <0.001 |
| SHAM | t0 – t1 | 32.5 | 102.8 | 0.32 | 1.000 |
| PAR IPSI | t0 – t1 | 467.4 | 112.5 | 4.15 | <0.001 |
| PAR CONTRA | t0 – t1 | -66.5 | 95.7 | 0.70 | 1.000 |

Note. For the sake of readability, only pre-post comparisons within each tDCS session are reported. OCC IPSI: ipsilesional occipital tDCS; PAR IPSI: ipsilesional parietal tDCS; PAR CONTRA: contralesional parietal tDCS.

# Differences between tDCS responders and non-responders

Differences between tDCS responders and non-responders (based on post-tDCS RTs changes on EF and Triangles Tasks) in demographic, clinical and lesional variables, as well as baseline (t0) performance. Mann-Whitney tests were performed. Results are reported in the Supplementary Tables 22, 23, and 24

**Supplementary Table 22. Differences between responders and non-responders to ipsilesional occipital tDCS**

|  | **EF Task** |  | **Triangles Task** |  |
| --- | --- | --- | --- | --- |
| **Variable** | **Statistics** | ***p*** | **Statistics** | ***p*** |
| Age | 17.50 | 1.000 | 12.50 | 0.423 |
| Disease Duration | 9.50 | 0.199 | 2.00 | 0.013 |
| Baseline t0 – EF Task | 11.00 | 0.310 | 11.00 | 0.310 |
| Baseline t0 – Triangles Task | 18.00 | 1.000 | 8.00 | 0.132 |
| Volume | 16.00 | 0.818 | 8.00 | 0.132 |
| Occipital Lobe | 15.00 | 0.688 | 6.00 | 0.065 |
| Parietal Lobe | 12.00 | 0.347 | 4.50 | 0.026 |
| Temporal Lobe | 13.50 | 0.514 | 8.50 | 0.142 |
| Temporo-parietal Lobe | 18.00 | 1.000 | 7.50 | 0.103 |
| Calcarine | 13.00 | 0.470 | 7.00 | 0.092 |
| Cuneus | 8.50 | 0.124 | 11.00 | 0.267 |
| Lingual | 18.00 | 1.000 | 9.00 | 0.173 |
| Occipital - Sup | 11.50 | 0.328 | 10.50 | 0.254 |
| Occipital - Mid | 17.00 | 0.935 | 14.50 | 0.625 |
| Occipital - Inf | 15.00 | 0.678 | 17.00 | 0.934 |
| Fusiform | 18.00 | 1.000 | 13.00 | 0.455 |
| Cingulum | 10.00 | 0.213 | 6.00 | 0.056 |
| Cingulum – anterior | 13.00 | 0.442 | 8.00 | 0.104 |
| Cingulum - posterior | 12.00 | 0.361 | 6.00 | 0.056 |
| Corpus Callosum | 12.00 | 0.394 | 7.00 | 0.093 |
| Fronto Striatal | 13.50 | 0.494 | 17.50 | 1.000 |
| IFOF | 14.00 | 0.589 | 12.00 | 0.394 |
| ILF | 17.00 | 0.936 | 12.00 | 0.378 |
| Optic Radiations | 16.00 | 0.818 | 16.00 | 0.818 |
| Pons | 13.50 | 0.494 | 17.50 | 1.000 |
| SLF II | 13.00 | 0.442 | 3.50 | 0.017 |
| SLF I | 12.00 | 0.347 | 4.50 | 0.026 |

*Note*: results of Mann-Whitney tests are reported. Sup: superior; Mid: middle; Inf: Inferior; IFOF: inferior fronto-occipital fasciculus; ILF: inferior longitudinal fasciculus; SLF I and SLF II: first and second branch of the superior longitudinal fasciculus.

**Supplementary Table 23. Differences between responders and non-responders to ipsilesional parietal tDCS**

|  | **EF Task** |  | **Triangles Task** |  |
| --- | --- | --- | --- | --- |
| **Variable** | **Statistics** | ***p*** | **Statistics** | ***p*** |
| Age | 14.50 | 0.630 | 15.0 | 0.688 |
| Disease Duration | 15.50 | 0.748 | 14.0 | 0.575 |
| Baseline t0 - EF | 16.00 | 0.818 | 13.0 | 0.485 |
| Baseline t0 - Tri | 18.00 | 1.000 | 13.0 | 0.485 |
| Volume | 14.00 | 0.589 | 18.0 | 1.000 |
| Occipital Lobe | 13.50 | 0.521 | 15.5 | 0.748 |
| Parietal Lobe | 15.50 | 0.732 | 16.5 | 0.864 |
| Temporal Lobe | 14.50 | 0.625 | 10.5 | 0.254 |
| Temporo-parietal Lobe | 15.50 | 0.744 | 14.0 | 0.568 |
| Calcarine | 16.50 | 0.873 | 16.5 | 0.873 |
| Cuneus | 13.50 | 0.494 | 16.5 | 0.864 |
| Lingual | 17.50 | 1.000 | 15.5 | 0.748 |
| Occipital - Sup | 10.00 | 0.222 | 15.0 | 0.684 |
| Occipital - Mid | 15.00 | 0.684 | 16.0 | 0.807 |
| Occipital - Inf | 15.00 | 0.678 | 17.0 | 0.934 |
| Fusiform | 14.00 | 0.561 | 14.0 | 0.561 |
| Cingulum | 10.00 | 0.213 | 17.0 | 0.934 |
| Cingulum – anterior | 13.00 | 0.442 | 15.0 | 0.669 |
| Cingulum - posterior | 9.00 | 0.158 | 15.0 | 0.678 |
| Corpus Callosum | 13.00 | 0.485 | 16.0 | 0.818 |
| Fronto Striatal | 16.50 | 0.864 | 16.5 | 0.864 |
| IFOF | 17.00 | 0.937 | 16.0 | 0.818 |
| ILF | 14.50 | 0.630 | 14.5 | 0.630 |
| Optic Radiations | 11.00 | 0.310 | 17.0 | 0.937 |
| Pons | 17.50 | 1.000 | 16.5 | 0.864 |
| SLF II | 14.50 | 0.608 | 16.5 | 0.864 |
| SLF I | 11.00 | 0.267 | 16.5 | 0.864 |

*Note*: results of Mann-Whitney tests are reported. Sup: superior; Mid: middle; Inf: Inferior; IFOF: inferior fronto-occipital fasciculus; ILF: inferior longitudinal fasciculus; SLF I and SLF II: first and second branch of the superior longitudinal fasciculus.

**Supplementary Table 24. Differences between responders and non-responders to contralesional parietal tDCS**

|  | **EF Task** |  | **Triangles Task** |  |
| --- | --- | --- | --- | --- |
| **Variable** | **Statistics** | ***p*** | **Statistics** | ***p*** |
| Age | 10.00 | 0.229 | 12.50 | 0.423 |
| Disease Duration | 6.00 | 0.065 | 11.50 | 0.336 |
| Baseline t0 - EF | 13.00 | 0.485 | 12.00 | 0.394 |
| Baseline t0 - Tri | 6.00 | 0.065 | 11.00 | 0.310 |
| Volume | 7.00 | 0.092 | 15.50 | 0.748 |
| Occipital Lobe | 14.50 | 0.608 | 15.50 | 0.732 |
| Parietal Lobe | 5.50 | 0.050 | 9.50 | 0.192 |
| Temporal Lobe | 12.00 | 0.370 | 10.50 | 0.254 |
| Temporo-parietal Lobe | 6.00 | 0.065 | 12.00 | 0.394 |
| Calcarine | 14.00 | 0.575 | 13.50 | 0.521 |
| Cuneus | 16.50 | 0.864 | 17.50 | 1.000 |
| Lingual | 6.00 | 0.065 | 14.50 | 0.630 |
| Occipital - Sup | 12.00 | 0.370 | 17.00 | 0.935 |
| Occipital - Mid | 10.50 | 0.254 | 17.00 | 0.935 |
| Occipital - Inf | 9.00 | 0.158 | 9.00 | 0.158 |
| Fusiform | 7.00 | 0.081 | 9.00 | 0.158 |
| Cingulum | 8.00 | 0.115 | 18.00 | 1.000 |
| Cingulum – anterior | 12.00 | 0.347 | 16.50 | 0.864 |
| Cingulum - posterior | 8.00 | 0.115 | 14.00 | 0.561 |
| Corpus Callosum | 6.00 | 0.065 | 15.00 | 0.699 |
| Fronto Striatal | 17.50 | 1.000 | 8.00 | 0.104 |
| IFOF | 5.00 | 0.041 | 9.00 | 0.180 |
| ILF | 6.00 | 0.065 | 8.50 | 0.149 |
| Optic Radiations | 6.00 | 0.065 | 11.00 | 0.310 |
| Pons | 17.50 | 1.000 | 8.00 | 0.104 |
| SLF II | 15.50 | 0.732 | 17.50 | 1.000 |
| SLF I | 10.00 | 0.200 | 16.00 | 0.798 |

*Note*: results of Mann-Whitney tests are reported. Sup: superior; Mid: middle; Inf: Inferior; IFOF: inferior fronto-occipital fasciculus; ILF: inferior longitudinal fasciculus; SLF I and SLF II: first and second branch of the superior longitudinal fasciculus.

# Exploratory differences between left- and right-brain damaged participants

Mann-Whitney comparisons of demographic, clinical and lesional, variables are reported in the Supplementary Table 25, whereas differences in post-tDCS RTs changes can be found in the Supplementary Table 26

**Supplementary Table 25. Differences between left- and right-brain damaged participants in demographic, clinical, and lesional variables**

| **Variable** | **z** | **P** |
| --- | --- | --- |
| Age | 21.50 | 0.684 |
| Disease Duration | 18.00 | 0.435 |
| Volume | 17.00 | 0.684 |
| Occipital Lobe | 15.50 | 0.729 |
| Parietal Lobe | 14.50 | 0.744 |
| Temporal Lobe | 13.50 | 0.501 |
| Temporo-parietal Lobe | 15.00 | 0.400 |
| Calcarine | 14.50 | 0.321 |
| Cuneus | 12.50 | 0.508 |
| Lingual gyrus | 14.50 | 0.312 |
| Occipital Sup | 11.00 | 0.238 |
| Occipital_Mid | 13.00 | 0.807 |
| Occipital Inf | 11.00 | 0.665 |
| Fusiform | 10.00 | 0.563 |
| Corpus Callosum | 14.00 | 0.290 |
| IFOF | 13.00 | 0.603 |
| ILF | 10.50 | 0.435 |
| Optic Radiations | 7.00 | 0.225 |

Note: Sup: superior; Mid: middle; Inf: inferior; IFOF: Inferior Fronto-Occipital Fasciculus; ILF: Inferior longitudinal fasciculus.

**Supplementary Table 26. Differences between left- and right-brain damaged participants in RTs change scores**

| **Task** | **ACC/RTs** | **tDCS** | **z** | **P** |
| --- | --- | --- | --- | --- |
| **Visual Detection Task** | **ACC Blind** | **Occ Ipsi** | 18 | 0.491 |
|  |  | **Par Ipsi** | 17 | 0.414 |
|  |  | **Par Contra** | 15.5 | 0.301 |
|  |  | **Sham** | 11 | 0.108 |
|  | **ACC Sighted** | **Occ Ipsi** | 21.5 | 0.795 |
|  |  | **Par Ipsi** | 14 | 0.190 |
|  |  | **Par Contra** | 18 | 0.468 |
|  |  | **Sham** | 11 | 0.108 |
|  | **RTs Blind** | **Occ Ipsi** | 16 | 0.345 |
|  |  | **Par Ipsi** | 13 | 0.181 |
|  |  | **Par Contra** | 20 | 0.662 |
|  |  | **Sham** | 9 | 0.059 |
|  | **RTs Sighted** | **Occ Ipsi** | 10 | 0.081 |
|  |  | **Par Ipsi** | 15 | 0.282 |
|  |  | **Par Contra** | 20 | 0.662 |
|  |  | **Sham** | 15 | 0.282 |
| **EF Task** | **ACC** | **Occ Ipsi** | 22 | 0.836 |
|  |  | **Par Ipsi** | 23.5 | 1 |
|  |  | **Par Contra** | 21 | 0.734 |
|  |  | **Sham** | 19.5 | 0.581 |
|  | **RTs** | **Occ Ipsi** | 23 | 0.950 |
|  |  | **Par Ipsi** | 2 | 0.003 |
|  |  | **Par Contra** | 23 | 0.950 |
|  |  | **Sham** | 22 | 0.852 |
| **Triangles Task** | **ACC** | **Occ Ipsi** | 12 | 0.132 |
|  |  | **Par Ipsi** | 24 | 1 |
|  |  | **Par Contra** | 18.5 | 0.514 |
|  |  | **Sham** | 21.5 | 0.795 |
|  | **RTs** | **Occ Ipsi** | 8 | 0.043 |
|  |  | **Par Ipsi** | 10 | 0.081 |
|  |  | **Par Contra** | 16 | 0.345 |
|  |  | **Sham** | 19 | 0.573 |

Note: RTs: response times; ACC: accuracy; Blind: blind hemifield; Sighted: sighted hemifield. Occ Ipsi: ipsilesional occipital tDCS; Par Ipsi: ipsilesional parietal tDCS; Par Conra: contralesional parietal tDCS.
